# Comparison of Neuroprotective Effects of *Melissa officinalis* Total Extract and Its Acidic and Non-Acidic Fractions against A β-Induced Toxicity

**Published:** 2013

**Authors:** Mohammad Reza Sepand, Maliheh Soodi, Homa Hajimehdipoor, Masoud Soleimani, Ehsan Sahraei

**Affiliations:** a *Department of Toxicology, School of Medical Sciences, Tarbiat Modares University, Tehran, Iran. *; b *Department of Traditional Pharmacy, School of Traditional Medicine, Shahid Beheshti University of Medical Sciences, Tehran, Iran. *; c *Department of Hematology, Faculty of Medical Sciences, Tarbiat Modares University, Tehran, Iran.*

**Keywords:** Beta-Amyloid, *Melissa officinalis*, Fractions, Neuroprotection, Oxidative stress

## Abstract

Alzheimer’s disease (AD) is a neurodegenerative disease that was characterized with deposit of beta amyloid (A*β*) aggregate in senile plaque. Oxidative damage to neurons and loss of cholinergic neurons in forebrain region are observed in this disease. *Melissa officinalis* is a medicinal plant from Lamiaceae family, used traditionally in the treatment of cognitive disorders. It has cholinomimetic and potent antioxidant activity. In the present study, we investigated the possible neuroprotective effects of total ethanolic extract, acidic and nonacidic fraction of *Melissa officinalis *on A*β*-induced cytotoxicity and oxidative stress in PC12 cells and also measured their *in-vitro *anticholinesterase activity. PC12 cells were incubated with the extract and fractions prior to the incubation with A*β *and cell toxicity was assessed by MTT assay. In addition, productions of reactive oxygen species (ROS), Malondialdehyde (MDA) as a biomarker of lipid peroxidation and glutathione peroxidase activity were measured. Pretreatment of cells with total extract and acidic fraction (not non-acidic fraction) had protective effect against A*β*-induced oxidative changes and cell death. In concentrations in which both total extracts of an acidic fraction showed neuroprotective effects, inhibition of cholinesterase activity was not significant. Then, the protective effects of *Melissa officinalis* total extract and acidic fraction were not attributed to their anticholinesterase activity. Acidic fraction showed more potent protective effect compared to the total extract, leading to the fact that polyphenolic compounds and terpenoic acids are the most effective components in the total extract concentrated in this fraction.

## Introduction

Alzheimer’s disease (AD) is a type of degenerative disease of the central nervous system in elderly patients. Neurodegenerative disorder was characterized by cognitive disabilities with severe dementia, memory deficiency, abstract thinking and personality alteration. (1). Two hallmarks in the pathogenesis of AD are neurofibrillary tangles (NFT) and neuritic plaques. The major component of plaques, which are extracellular deposits, is aggregated fibrillar *β*-amyloid (A*β*) peptide (2). Several microscopic studies of post-mortem human brain tissue of AD patients suggest that extracellular deposition of A*β *peptides plays the most important role in AD pathogenesis (3). A*β *peptide is generated from *β*-amyloid precursor protein (APP) by sequential cleavage through *β*-secretase and *γ*-secretase. Abnormal metabolism of APP results in increased production, accumulation and deposition of A*β *and lead to neuronal cell death in AD (4). Several mechanisms that proposed to explain the neurotoxicity of A*β* includes: production of reactive oxygen species (ROS) and oxidative stress, mitochondrial abnormalities and depletion of cellular ATP, elevated intracellular calcium and excitotoxicity and induction of inflammatory responses. All this mechanisms result in synaptic dysfunction and finally neuronal loss through activation of apoptotic and necrotic cell death pathways (2).

 Extensive evidence suggests that the oxidative stress plays an important role in pathogenesis of AD. Oxidative damage induced by ROS, including protein, DNA, RNA oxidation and lipid peroxidation, have been described in the AD brain. It is supposed that the A*β *toxicity, at least partially, is induced by free radicals (5).

Therefore, therapeutic effort for attenuating the free radicals or preventing their production could be beneficial in AD treatment. Hence, antioxidants may be merged as one of therapeutic strategies to attenuate A*β*-induced neurotoxicity and improve neurological outcome in AD (6, 7).

Moreover, gradually loss of cholinergic neurons in basal forebrain is contributed with AD pathology via memory deficiency (8). Drugs approved for AD treatment are acetylcholinesterase (AChE) inhibitors which can improve cognitive impairment but cannot prevent disease progression (9). Because of complex pathology of AD, new attempts are going to produce therapeutic agent that can halt disease progress through different pathways (10). Herbal medicines pose several components with different pharmacological effects and may be more effective in complex diseases. In recent years, several studies were considered as therapeutic effects of herbal medicines in AD therapy (11).

*Melissa officinalis *(lemon balm) is a medicinal plant from Lamiaceae family. It has been used as folk medicine for long time in Iran (12). Medicinal preparations of this herb were used for treatment of indigestion, anemia, palpitation and mood disorders (13). *M. officinalis *has effect on nervous disorders including the reduction of excitability, anxiety and stress, and sleep disturbance (14). Moreover, *M. officinalis *has neurotropic activity (15). Total extract of *M. officinalis *and different fractions of it have anticholinesterase activity (16). *M.* *officinalis *extract shows the potent antioxidant activity and plant extracts could protect cells against oxidative damage induced by different pro-oxidant agents which eventually leads to lipid peroxidation by different process (17). Furthermore, the administration of *M. officinalis* extract in AD patients can improve symptoms of disease (18). However, the mechanism and constituents involved in its neuroprotective properties are not well known. It is claimed that the main effective components of this plant are polyphenols and terpenoid compounds. The aim of our study was to investigate and compare the neuroprotective effect of total ethanolic extract, acidic fraction which contains polyphenols and non-acidic fraction which is free of polyphenols. In addition, the effects of the extracts on oxidative stress biomarkers and cholinesterase activity were studied as well.

## Experimental

*Materials*

Rat pheochromocytoma (PC12) cell line was purchased from national cell bank of Iran (NCBI, Pasteur Institute of Iran). A*β*(25-35 ),2′7′-dichlorofluorescin diacetate (DCFHDA), 3-(4,5-dimethylthiazol-2-yl)-2,5-diphenyl tetrazolium bromide (MTT), poly-D-lysin (PDL), Glutathione Peroxidase (GSH-Px) activity assay kit, Acetylcholinesterase (AChE, Type V-S, lyophilized powder, 1000 unit/mg protein), 5,5-Dithiobis-(2-nitrobenzoic acid) (DTNB), acetylthiocholine iodide, Malondialdehyde bis (dimethyl acetal) and Thiobarbituric acid were purchased from Sigma. RPMI 1640 medium, penicillin-streptomycin and fetal bovine serum (FBS) were purchased from Gibco.

*Plant material*

The leaves of *Melissa officinalis *were collected from Gorgan (Golestan province) in Jun 2009 and identified by M. Kamalinejad, botanist from Faculty of Pharmacy, Shahid Beheshti University of Medical Sciences. A voucher specimen was kept in Herbarium of Faculty of Pharmacy, SBMU, Tehran, Iran (NO. 545).

*Plant extraction*

Total plant extract was obtained by the extraction of dried and milled plant leaves with ethanol 80% (1:10) by using maceration method for 4 days. After every 24 h, the mixture was filtered and new solvent was added to the plant powder. The combined extracts were concentrated to dryness.

 In order to prepare acidic fraction of the plant, 50 mL of NaOH 0.1 N was added to 4 g of plant total extract and mixed. The aqueous phase was separated and this process was repeated for two more times. Nonaqueous phase contained nonacidic fraction. Aqueous phase was acidified with HCl 1 N and extracted with ethyl acetate for three times. The combined ethyl acetate layers were concentrated under vacuum pressure to dryness (acidic fraction) (19).

*Cell culture and treatment*

PC12 cells were cultured on PDL-coated flasks containing RPMI 1640, supplemented with 10% (v/v) heat-inactivated fetal bovine serum, and 1% (v/v) penicillin and streptomycin. Cultures were maintained at 37°C in a humidified atmosphere containing 5% CO_2_. These cells were seeded at appropriate densities on PDL coated 96- well plates for viability assay or 6-well plates for oxidative stress biomarkers assay. Twenty-four hours after the seeding, cells were preincubated with different concentrations of total extract (0.1-100 μg/mL), acidic fraction (0.001-10 μg/ mL) and non-acidic fraction (0.01-10 μg/mL) for 1 h and incubated with 20μM A*β *peptide for additional 24 h. Stock solution of total extract and acidic fraction were prepared in PBS and further diluted with the medium to appropriate concentrations. Non-acidic fraction was

dissolved in DMSO and diluted with the medium to proper concentrations. Final concentration of

DMSO was 0.1% in medium. Stock solution of A*β *peptide (1 mM) was prepared by dissolving 1 mg in 1 mL sterile distillated water and stored in - 80°C until use. Prior to use, A*β *peptide was

aggregated for 3 days in 37°C.

*Cell viability assay*

PC12 cells were plated in PDL-coated 96-well plates (10^4^ cells/well) and incubated with A*β *peptide with or without different concentrations of total extract and fractions as described above for 24 h. After the incubation, the medium was replaced with fresh medium containing MTT solution (final concentration 0.5 mg/mL) and incubated for 4 h in 37°C. Then, the medium was removed and 100 μL DMSO was added to each well and mixed properly until the blue formazan product completely dissolved. Absorbance was measured at 540 nm in an automated plate reader (BIOTEK) against 670 nm as the reference wavelength. Results were reported as the percentage of control group (20).

*Measurement of lipid peroxidation*

Malondialdehyde (MDA), the most abundant lipid peroxidation product from PC12 cells, was measured using the thiobarbituric acid (TBA) colorimetric assay. PC12 cells were plated in PDL-coated 6-well plates (10^6^ cells/ well) and incubated with A*β *peptide with or without total extract (10 μg/mL) and acidic fraction (1 μg/mL) as described above for 24\ h. After the incubation, cells were washed with PBS, and then harvested with 1 mL icecold PBS containing 0.5 mM EDTA and 1.13 mM butyl-hydroxytoluene and sonicated for 20 sec. Twenty μL of cell lysate were removed for protein analysis. One volume of cell lysate was mixed with two volume of TBA reagent (containing 3.75% TCA and 0.0925% TBA) and the mixture was incubated at 90°C

for 60 min. After cooling, the mixture was centrifuged at 1000 g for 10 min and the optical density of supernatant was measured in 540 nm in plate reader. MDA standard curve was established using the stable MDA precursor, Malondialdehyde bis (dimethyl acetal). The results are presented as micromole of MDA/ microgram protein (21, 22). The amount of protein was measured by Bradford method (23).

*Measurement of ROS production*

The cellular ROS was quantified as described by Hong *et al. *(24). The accumulation of intracellular ROS can be detected by using DCFH-DA, which crosses cell membranes and is hydrolyzed enzymatically by intracellular esterases to nonfluorescent dichlorofluorescein (DCFH) that is often used as an indicator of ROS. In the presence of ROS, DCFH is oxidized to highly fluorescent dichlorofluorescein (DCF). After the incubation of PC12 cells with A*β *peptide with or without total extract (10 μg/ mL) and acidic fraction (1μg/mL), the cells were harvested by trypsinization and incubated with 10 μmol DCFH-DA in PBS containing 5.6 mmol glucose at 37ºC for 40 min and then centrifuged and suspend in 1 mL PBS buffer. The fluorescence intensity was measured by flowcytometry (BD, U.S.A.) at an excitation wavelength of 488 nm and an emission wavelength of 525 nm.

*Glutathione peroxidase activity*

PC12 cells were seeded in PDL coated 6-well plates (10^6^ cells/well) and incubated with A*β *peptide with or without total extract (10 μg/mL) and acidic fraction (1 μg/mL) as described above for 24 h. After the incubation, cells were washed with PBS and harvested with trypsinization and homogenated in PBS. The homogenate was centrifuged at 1000 g for 10 min and supernatant was used for enzyme activity and protein assay. The activities of GSH-Px were measured by using the assay kits in accordance with the instructions supplied by the manufacturers.

*In-vitro acetylcholinesterase activity assay*

AChE activity assays were carried out using an acetylthiocholine iodide substrate-based colorimetric method, as described by Ellman (25). Briefly, 3 mL phosphate buffer (pH = 8), 100.0 μL Dithiobisnitrobenzoic acid (DTNB) 0.01 M as reagent, 50 μL AChE enzyme (3IU) and 50 μL extract were mixed and immediately after adding, 20 μL acetylthiocholine iodide (0.075 M) as a substrate, changes in absorbance at 412 nm was measured by spectrophotometer, in 30 sec interval during 6 min. a blank containing all components except AChE was run in parallel with sample in order to delete the spontaneous and non-enzymatic break down of acetylthiocholine. The reaction rates were calculated, and the percent inhibition of test compounds was determined.

*Statistical analysis*

All data were represented as the mean ± SE of three separate experiments. Statistical differences were estimated by using oneway ANOVA followed with Newman-Keuls Multiple Comparison Test.

## Results

*Cell viability*

The protective effect of *M. officinalis *total extract and acidic and non-acidic fractions were evaluated by MTT assay. Twenty-four hours treatment of PC12 cells with A*β *significantly reduced the cell viability. Pretreatment of cells for 1 h with different concentration of total extract and acidic fraction before adding A*β *peptide protected them from A*β*-induced toxicity (Figures 1A and B). These effects were dose-dependent 0.1 μg/mL of total extract and 0.001 μg/mL acidic fraction could slightly increase the cell viability, however, 10 μg/mL of total extract and 1 μg/mL of acidic fraction completely reversed the A*β*-induced toxicity and increased cell viability up to control level. As represented in Figure 1C, pretreatment of cells with non-acidic fraction could not protect them from A*β*-induced toxicity. Treatment with total extract (0.1-100 μg/mL), acidic fraction (0.001-10 μg/mL) and non-acidic fraction (0.01-10 μg/mL) alone did not decrease the cell viability compared to the control group (data not shown).

**Figure:**
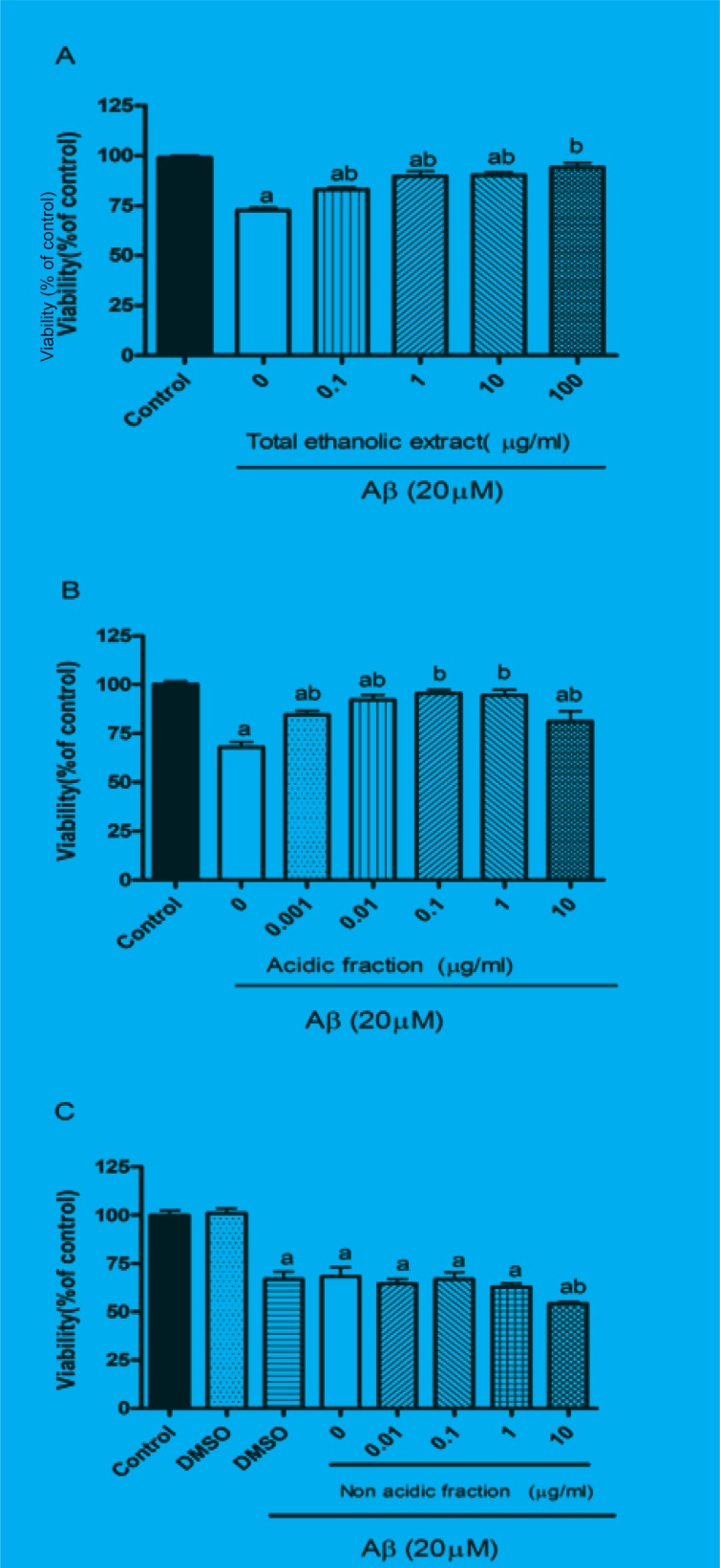
Effects of *M. officinalis *total extract (A), acidic fraction (B) and non-acidic fraction(C) on A*β*-induced toxicity in PC12 cells.

All data represent the mean ± SEM of three independent experiments. (a; represents different from control groups, b; represents different from A*β*-treated groups).

*Effect of M. officinalis total extract and acidic fraction on lipid peroxidation*

**Figure 2:**
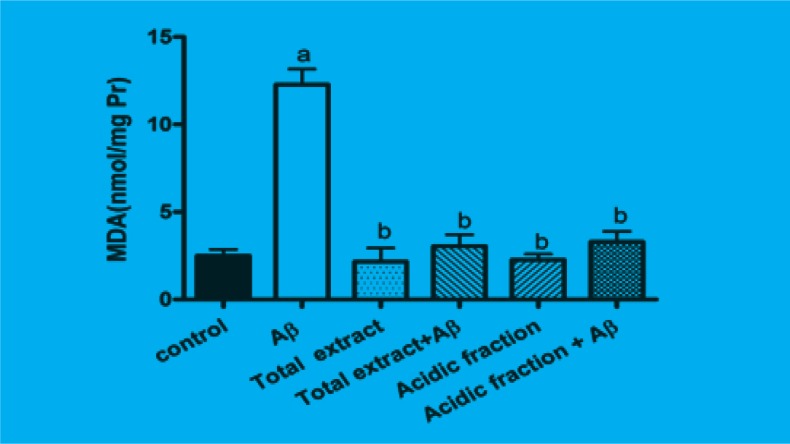
Inhibitory effects of *M. officinalis *total extract and acidic fraction on A*β*-induced lipid peroxidation in PC12 cells.

The amount of intracellular MDA which is the product of lipidperoxidation was significantly increased when cells were incubated for 24 h with A*β*. Pretreatment of cells with 10 μg/mL of total extract and 1 μg/mL of acidic fraction significantly attenuated the A*β*-induced MDA production. Treatment of cells with 10 μg/mL of total extract and 1 μg/mL of acidic fraction alone has not any effect on MDA production (Figure 2).

Data represent the mean ± SEM of three independent experiments. (a: represents different from control groups, b: represents different from A*β*-treated groups).

*Effect of M. officinalis total extract and acidic fraction on GSH-Px activity*

**Figure 3:**
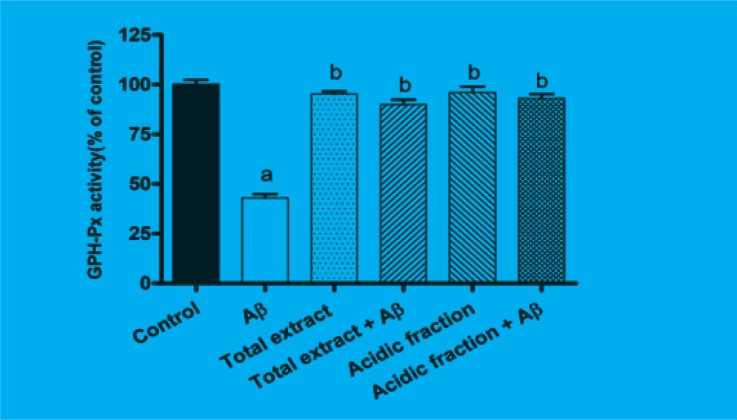
Effects of *M. officinalis *total extract and acidic fraction on A*β*-induced changes on GSH-Px activity in PC12.

GSH-Px is the most important antioxidant enzyme in cells. As shown in Figure 3, after 24 h of incubating the cells with A*β*, GSH-Px activity was significantly reduced as compared to the control group (p < 0.001). Pretreatment with 10 μg/mL of total extract and 1 μg/mL of acidic fraction attenuated the changes in GSHPx activity induced by A*β *treatment.

Data represent the mean ± SEM of three independent experiments. (a: represents different from control groups, b: represents different from A*β*-treated groups).

*Effect of M. officinalis total extract and acidic fraction on ROS production*

The treatment of PC12 cells with A*β *was found to increase the intracellular ROS levels (19). Exposure of PC12 cells to A*β *caused an increase in DCF fluorescence which is indicator of ROS production. When the cells were pretreated with 10 μg/mL of total extract and 1 μg/mL of acidic fraction before the addition of A*β*, the mean fluorescence intensities were lower than that of A*β *treatment group (Figure 4).

*Effect of M. officinalis total extract and acidic fraction on AChE activity*

Table 1 represents the percentage of enzyme activity inhibition in present of different concentrations of total extract and acidic fraction. Both total extract and acidic fraction inhibited enzyme activity about 50% in high concentrations (500 μg/mL); no enzyme activity inhibition was observed for 10 μg/mL of total extract and 1 μg/mL of acidic fraction.

**Table 1:** Inhibitory Activities of *M. officinalis *total extract and acidic fraction against AChE activity *in-vitro*.

**Compounds**	**Concentrations(μg/mL)**	**Inhibition of AChE activity (%)**
*M. Officinalis *total extract	10	N*
	100	21.08 ± 2.5
	500	40.47 ± 3.2
Acidic fraction	10	N
	100	33.25 ± 1.98
	500	53.51 ± 1.7

* No inhibitory effect was observed. Each value represents the mean ± SEM (n = 5)

## Discussion

PC12 cells have been widely used as an experimental model to study cellular neurotoxicity (27). Studies have shown that A*β*25-35 induced cytotoxicity in PC12 cells, which was accompanied with excessive ROS production, mitochondrial dysfunction (28) apoptosis and cell death (29), therefore, several studies use PC12 cells for studying agents against A*β *toxicity. Results of our study show that *M. officinalis *total extract can attenuate the A*β*-induced toxicity and oxidative stress. This plant has potent antioxidant activity with direct free radical scavenging activity, and it is reported that *M. officinalis *total extract can protect PC12 cells against hydrogen peroxide-induced cell death and oxidative stress (30). Oxidative stress is the mechanism that was assumed

for A*β *toxicity and Alzheimer’s etiology (6). Antioxidant can ameliorate disease progression (7), so it is supposed that antioxidant activity may be contributed to neuroprotective effect of *M. officinalis against *A*β *toxicity. In addition, we showed that the acidic fraction of extract is potent

and the total extract and non-acidic fraction have not protective effect. These findings led us to this fact that the effective components of extract were concentrated in acidic fraction. These components are polyphenols, flavonoids and terpenoids. The polyphenols that were found in *M. officinalis *extract are Caffeic acid derivatives and among them, rosmarinic acid is the major polyphenol component. Several biological activities have been described for rosmarinic acid, including antioxidative, anti-inflammatory, anti-mutagenic, antibacterial and antiviral activities (31). Rosmarinic acid is a potent antioxidant with direct free radical scavenging activity. Neuroprotective activity was also reported for this compound. It is reported that rosmarinic acid can protect A*β*-induced memory impairment in mice and this effect is due to the direct peroxynitrite scavenging activity (32). Moreover, pretreatment of PC12 cells with rosmarinic acid can protect them from A*β*- induced toxicity. It is suggested that rosmarinic acid inhibits oxidative stress and apoptosis (33). Another property reported for rosmarinic acid in literature is anti-acetylcholinesterase activity. Dastmalchi and *et al. *studied the anticholinesterase activity of *M officinalis *ethanolic extracts and fractions; they reported that one fraction of extract which contains rosmarinic acid has high anti-cholinesterase activity even more than total ethanolic extract (16).

The caffeic acid, a cinnamic acid derivative, and the ursolic acid, a triterpenoid, may contribute

with protective activity of *M. officinalis*. The neuroprotective effects for these compounds were

reported. Ursolic acid and Caffeic acid can rescue A*β*-induced oxidative stress in PC12 cells (34,

35). Ursolic acid is a triterpenoid with hydroxyl radical scavenging activity and it increases the activities of antioxidant enzymes such as superoxide dismutase, catalase and glutathione peroxidase (34). Besides, ursolic acid isolated from oregano (*Origanum majorana *L.) inhibited the enzyme acetylcholinesterase (35).

 In the present study, we assessed the effect of total extract and acidic fraction on acetylcholine esterase activity. We found that both total extract and acidic fraction could inhibit acetylcholine esterase activity and acidic fraction was more potent compared to the total extract, which was in agreement with the previous study as rosmarinic acid is concentrated in acidic fraction. But this effect was observed in high concentrations and in concentrations which total extract and acidic fraction had protective effect against A*β*- induced toxicity. Anti-cholinesterase activity was not observed in the total extract and acidic fraction, leading to the fact that protective effect of extract and acidic fraction were not attributed to inhibition of cholinesterase enzyme.

 It is reported that the *M. officinalis *extract contains compounds with acetylcholine receptor affinities and that the affinity for nicotinic receptor is more than muscarinic receptor. IC_50_-values for [^3^H] nicotine displacement for ethanolic extract of *M. officinalis *were lower than 100 μg/mL (36). Several studies have been performed to show the role of nicotinic receptors in A*β*-induced toxicity. *In-vitro *studies showed that nicotine has protective effect on A*β*-induced toxicity and preincubation of neurons with nicotine attenuated the A*β*-induced oxidative stress and apoptosis. The protective effect of nicotine was antagonized with mecamylamine, a nicotinic receptor antagonist. Results of these studies indicated that the nicotinic receptor stimulation can protect neurons against A*β*- induced toxicity (37, 38). According to these studies, it can be suggested that the protective effect of *M. officinalis *and acidic fraction against A*β*-induced toxicity may be exerted through the nicotinic receptors. The nature of compounds in *M. officinalis *extract which have nicotinic receptor affinity, were unknown. Our finding ruled out the existence of basic nitrogenous compounds, as these compounds, in case of existence, would be concentrated in basic fraction, and our result showed that the basic fraction of extract did not have any protective effect against the A*β*-induced toxicity.

## Conclusion

Our finding represents that the acidic fraction of *M. officinalis *has significant protective effect on A*β*-induced toxicity and oxidative stress. These effects do not contribute to the anti-acetylcholinesterase activity of extract. According to our finding and previous study, we concluded that the protective effect of this plant contribute to the polyphenols and triterpenoids

and may be exerted through antioxidant mechanism or nicotinic receptor stimulation. These hypotheses need further investigation, and we will study it in our future research.
